# Nutrition Management Miniprograms in WeChat: Evaluation of Functionality and Quality

**DOI:** 10.2196/56486

**Published:** 2024-09-12

**Authors:** Hui Sun, Yanping Wu, Jia Sun, Wu Zhou, Qian Xu, Dandan Hu

**Affiliations:** 1 School of Medicine, Southeast University, No.87 Dingjiaqiao, Hunan Road, Naning, 210003, China; 2 The Affiliated Lianyungang Hospital of Xuzhou Medical University, Lianyungang, China

**Keywords:** nutrition management, WeChat mini-program, User Version of the Mobile Application Rating Scale, uMARS, function and quality evaluation

## Abstract

**Background:**

With the rise in people’s living standards and aging populations, a heightened emphasis has been placed in the field of medical and health care. In recent years, there has been a drastic increase in nutrition management in domestic research circles. The mobile nutritional health management platform based on WeChat miniprograms has been widely used to promote health and self-management and to monitor individual nutritional health status in China. Nevertheless, there has been a lack of comprehensive scientific evaluation regarding the functionality and quality of the diverse range of nutritional miniprograms that have surfaced in the market.

**Objective:**

This study aimed to evaluate the functionality and quality of China’s WeChat nutrition management miniprogram by using the User Version of the Mobile Application Rating Scale (uMARS).

**Methods:**

This observational study involves quantitative methods. A keyword search for “nutrition,” “diet,” “food,” and “meal” in Chinese or English was conducted on WeChat, and all miniprograms pertaining to these keywords were thoroughly analyzed. Then, basic information including name, registration date, update date, service type, user scores, and functional scores was extracted from January 2017 to November 2023. Rating scores were provided by users based on their experience and satisfaction with the use of the WeChat miniprogram, and functional scores were integrated and summarized for the primary functions of each miniprogram. Moreover, the quality of nutrition management applets was evaluated by 3 researchers independently using the uMARS.

**Results:**

Initially, 27 of 891 miniprograms identified were relevant to nutrition management. Among them, 85.2% (23/27) of them offered features for diet management, facilitating recording of daily dietary intake to evaluate nutritional status; 70.4% (19/27) provided resources for nutrition education and classroom instruction; 59.3% (16/27) included functionalities for exercise management, allowing users to record daily physical activity; and only 44.4% (12/27) featured components for weight management. The total quality score on the uMARS ranged 2.85-3.88 (median 3.38, IQR 3.14-3.57). Engagement scores on the uMARS varied from 2.00 to 4.33 (median 3.00, IQR 2.67-3.67). Functional dimension scores ranged from 3.00 to 4.00 (median 3.33, IQR 3.33-3.67), with a lower score of 2.67 and a higher score of 4.33 outside the reference range. Aesthetic dimension scores ranged from 2.33 to 4.67 (median 3.67, IQR 3.33-4.00). Informational dimension scores ranged from 2.33 to 4.67 (median 3.33, IQR 2.67-3.67).

**Conclusions:**

Our findings from the uMARS highlight a predominant emphasis on health aspects over nutritional specifications in the app supporting WeChat miniprograms related to nutrition management. The quality of these miniprograms is currently at an average level, with considerable room for functional improvements in the future.

## Introduction

With the intensification of the population’s aging tendency, people pay increasing attention to the health of older adults. Specifically, nutrition management is of paramount importance for older adults, in that nutritional needs not only impact their quality of life but also significantly influence overall health status [1]. Aging, a normal process, is accompanied by physiological changes such as a loss of muscle mass, reduction in bone density, and decline in metabolic rate; Therefore, it is of vital importance to adjust nutritional intake among older individuals, especially those with weak digestive systems [2].

Malnutrition among older adults is a significant universal concern. Senile malnutrition, characterized by inadequate and imbalanced nutrition, arises from various factors, such as inappropriate dietary choices, insufficient intake, absorption disorders, etc [3]. The aging demographic shift contributes to a rise in the population of older adults, and the health status of the older adults directly impacts societal sustainability [4]. Consequently, there is an urgent need for effective nutrition policies and intervening measures for older adults. Among them, nutrition education, promotion of a balanced diet, optimization of medical and health care systems are essential strategies to prevent and improve senile malnutrition [5].

Thus far, the government has issued a series of policies, including the “Healthy China 2030 Plan Outline,” which emphasizes the promotion of self-disciplined health behaviors and encourages balanced diets. Concurrently, the National Nutrition Plan (2017-2030) advocates for the integration of “Internet+nutrition and health” [6], endorsing the use of technology to manage public health and nutrition. Nowadays, this shift toward digital health management and intelligent nutrition support system for older adults reassure the growing emphasis on leveraging science and technology in health care. Through internet platforms and mobile apps, the dietary habits of older adults can be more effectively monitored, and personalized dietary recommendations can be provided to identify and address possible malnutrition issues promptly [7]. WeChat, an instant messaging software for smart terminals, was launched by Tencent on January 21, 2011, and it emerged in 2017 with its distinct advantages, including user-friendly miniprograms that do not require downloading, occupy minimal mobile phone memory, and include payment capabilities, circles of friends, public platforms, WeChat miniprograms, and other functionalities. All these features have contributed greatly to its widespread adoption among users [8].

Despite the growing number of mobile apps for nutrition guidance, there is remarkable variability among them due to a lack of specificity. Studies in China have predominantly focused on exploring the effects of interventions and the significance of nutrition research; yet, there remains a notable absence of scientific evaluation regarding the functionality and quality of commercially available nutrition miniprograms [9,10]. Accordingly, this study aimed to carry out a comprehensive search and assessment of relevant miniprograms using the User Version of the Mobile Application Rating Scale (uMARS) and develop WeChat-based applets for nutrition management.

## Methods

### Search Strategy

A keyword search for “nutrition,” “diet,” “food,” and “meal” in Chinese or English was independently conducted on WeChat by 2 researchers, and all miniprograms pertaining to these keywords were thoroughly analyzed. They personally experienced the relevant miniprograms registered on WeChat between January 2017 and November 2023. Screening for miniprograms strictly adhered to predefined inclusion and exclusion criteria.

The inclusion criteria were as follows: (1) the miniprogram’s functional content pertained to diet and nutrition, (2) it was available for free use, (3) its content was written in Chinese or English, and (4) it was compatible with mobile phones or tablets. The exclusion criteria were as follows: (1) it was never updated or maintained, (2) it was designed for commercial ordering and canteen services, (3) it was specifically for purchasing food, (4) it solely records data. During the screening process, the 2 researchers resorted to another researcher, if necessary, to resolve any discrepancies.

### Sample Size

In this study, an observational research design was adopted to systematically search and quantitatively evaluate the function and quality of nutrition management miniprograms on WeChat. Using keywords such as “nutrition,” “diet,” “food,” and “meal,” a total of 891 miniprograms were initially identified. These miniprograms were then screened based on predefined inclusion and exclusion criteria. After the screening process, 27 eligible miniprograms were selected for detailed analysis.

### Quality Assessment of WeChat Miniprograms Related to Nutrition Management

As described in a previous study [11], the uMARS was originally developed as an end user evaluation tool based on the Mobile Application Rating Scale (MARS), and it has been widely used for evaluating diverse categories of mobile health apps, which include those focusing on weight loss and nutrition, as well as the management of conditions such as rheumatism and ankylosing spondylitis [12].

As a comprehensive tool for evaluating the user experience of mobile apps, the uMARS was commonly used to gauge usability, user satisfaction, functionality, and other pertinent factors [13]. The 5 dimensions of the uMARS are engagement, functionality, aesthetics, information, and subjective quality, each dimension encompassing 3‐5 questions and each question scored on a scale of 0-5 points (20 items in total) [14,15]. In the engagement dimension, the evaluator can evaluate the miniprogram’s entertainment value, level of interest, customization, interactivity, and target audience appeal. The functional dimension facilitates the evaluation of performance, ease of use, navigation, and gesture design. The aesthetics section focuses on layout, graphics quality, and visual appeal, and the information section assesses information quality, quantity visual information, and the credibility of the information source [16-18]. Notably, to ensure evaluation consistency, the subjective scale is not included in the assessment process due to the highly subjective nature of evaluators’ personal opinions and preferences.

### Participants

Evaluation of the uMARS was conducted independently by 2 nutrition experts and 1 experiencer. Before the evaluation, each evaluator was required to read and further familiarize themselves with dimensions and items of the scale. All evaluators must comply with the consensus of the scoring criteria reached by the group discussion and evaluate each applet independently.

### User Scores

User scores, a built-in feature of WeChat applets, are intended to assess users’ overall satisfaction and experience, and a 5-point satisfaction scale is commonly used to measure people’s satisfaction levels with the applets for research and surveys. Developers use these ratings to gauge the satisfaction levels of existing users and identify any areas for enhancement. If a feature of a WeChat miniprogram is not properly activated or if no users have participated in the rating, the default rating is set to 0.

### Functional Scores

From among the 27 miniprograms selected, 16 functions were identified on the basis of registration and usage. Each miniprogram’s functionality was evaluated by assigning 1 point for each aspect assessed, contributing to the cumulative total score. Feature scores ranged from 0 to 16, illustrating the comprehensiveness of the miniprograms’ features.

### Data Collection

Basic information regarding the 27 selected miniprograms was collected, encompassing metrics such as name, registration date, update date, service type, user scores, and functional scores. Subsequently, the functionality and quality of these miniprograms were quantitatively assessed using the uMARS.

### Statistical Analyses

The functional score of each miniprogram was collected and expressed as quantity and percentage values. uMARS scores were described as mean and SD or IQR values. All data were analyzed using SPSS (version 26.0; IBM Corp).

### Ethical Considerations

This study was approved by Zhongda Hospital affiliated to Southeast University (2024ZDSYLL194-P01).

## Results

### Screening of Miniprograms for Nutrition Management

**Figure 1.**
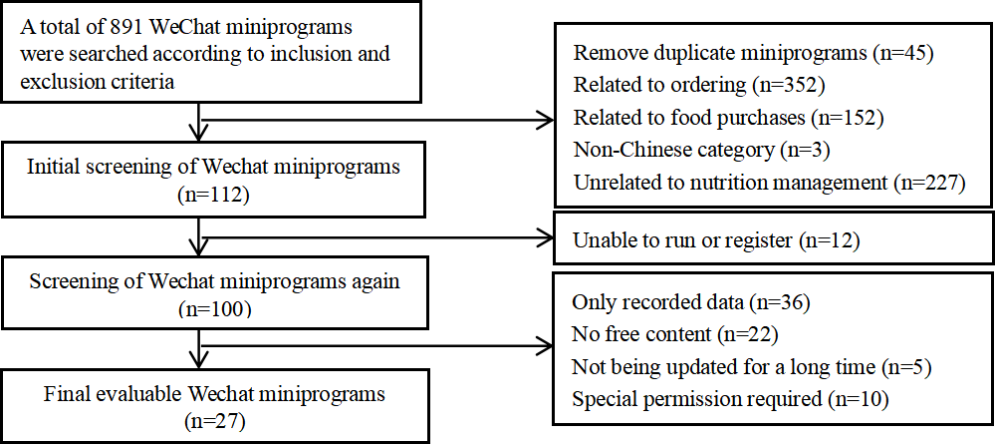
Flowchart for screening of nutrition management miniprograms.

We identified 891 miniprograms based on search terms (Figure 1). After removing the duplicate and unrelated miniprograms, 112 miniprograms were filtered. Ultimately, 27 miniprograms met the inclusion and exclusion criteria were selected for further study.

### Characteristics of Nutrition Management Miniprograms

We conducted a thorough search for miniprograms related to nutrition management through WeChat and used them after registration. Interestingly, these miniprograms shared several common characteristics, including name, registration date, update date, service type, user scores, and functional scores (Table 1).

**Table 1.** Characteristics of the nutrition- related WeChat mini-programs^a^.

Name	Registration date	Update date	Service type	User scores	Functional scores
Peppermint nutritionist	Nov 15, 2018	Feb 10, 2023	Health management, medical information, food and beverage, and health products	—^b^	10
YOU nutrition	Apr 17, 2020	Nov 3, 2023	Health management, health care products, food and beverage, drug information display, and medical equipment sales platform	5	9
The more accurate and nutritious the diet	Oct 12, 2021	Nov 2, 2023	Food and beverage and health products	4.6	6
Long light nutrition diet therapy	Jul 19, 2021	Nov 2, 2023	Web-based education, educational information services, and medical information	—	7
Nutrition pagoda	Sep 14, 2022	Mar 10, 2023	Health management	3.6	7
Little Ann dietitian	Jan 15, 2021	Nov 4, 2023	Health management and equipment management	4.6	6
Abbott Medical Nutrition Care	Sep 18, 2019	Oct 28, 2023	Health management	4.5	5
Peppermint nutrition Pro	Dec 10, 2018	Jan 10, 2023	Health management	3.7	4
Nutritionist world	Aug 12, 2022	Nov 2, 2023	Health care products and food and beverage	3.5	4
Nutritional meal companion	Jul 9, 2022	Oct 28, 2023	Catering information service	—	6
Better One Nutritional fat reduction	Jan 19, 2022	Oct 28, 2023	Beauty service	—	6
Nutrition weight loss service platform	Aug 28, 2023	Nov 4, 2023	Health management and web-based fitness	—	8
Carkaka |Meal control card assistant	Feb 17, 2023	Oct 26, 2023	Recipe drinks, community/forum, and health management	4.1	6
High uric acid diet	Oct 27, 2017	Nov 6, 2023	Information inquiry, health management, and community/forum	4.3	6
Food diary	Mar 8, 2020	Nov 1, 2023	Catering information service and medical information	4.7	3
AI Dietary dietitian	Dec 5, 2022	Nov 6, 2023	Information query, video customer service, and health management	—	6
Little Doctor’s diet diary	Nov 10, 2022	Jul 10, 2023	Health data statistics	—	5
High potassium diet	Dec 22, 2017	Nov 5, 2023	Information inquiry, health management, and community/forum	4.6	6
Chestnut food diary	Nov 27, 2018	Oct 28, 2023	Health management and medical information	4.3	8
Dietary calories	Oct 4, 2021	Sep 12, 2023	Information inquiry, food and beverage information service, and health management	4.2	5
Low carb diet assistant	Jun 21, 2019	Nov 12, 2022	Health management	4.4	4
Diet evaluation	Feb 28, 2023	Jun 12, 2023	Information, health management, and drug information display	—	2
Food notes	Feb 1, 2023	Sep 12, 2023	Health management	4.1	4
Sannuo Health	Sep 20, 2019	Nov 6, 2023	Medical information, community/forum, drug information display, and medical device manufacturer	—	9
Pick fruit health	May 27, 2020	Oct 13, 2023	Food and beverage, equipment management, medical equipment, health products, and health management	4.0	6
Mint Health	Apr 26, 2017	Oct 13, 2023	Catering information service and information inquiry	4.6	7
Peak Health Butler	Oct 11, 2022	Jul 13, 2023	Health management and information inquiry	4.2	5

a (1) Health management: managing health through lifestyle changes; (2) medical information: information about diseases and health; (3) food and beverage: information about food nutrients; (4) health products: goods for maintaining or improving well-being; (5) drug information display: platform for medication details and usage guidance; (6) medical equipment sales platform: marketplace for health care equipment transactions; (7) educational information services: to provide information resources for health purposes; (8) medical information: information related to health care and treatments; (9) equipment management: access basic medical equipment data on the web; (10) catering information service: information hub for dining options and nutrition; (11) beauty service: offerings for cosmetic and aesthetic treatments; (12) web-based fitness: exercise and wellness programs accessible via the internet; (13) recipe drinks: formulations for beverages with health benefits; (14) community/forum: platform for discussions and interactions among users; (15) video customer service: support assistance provided through video communication; (16) health data statistics: analysis and presentation of health-related data; and (17) medical device manufacturer: producer of health care equipment.

b Not applicable.

### WeChat Miniprogram for Nutrition Management

Following registration and use, the functional evaluation mainly focused on the nutrition management module of the miniprogram. This module encompassed 4 primary functions (diet management, weight management, exercise management, and nutrition education through class, video, or popular science content) and 12 auxiliary functions (Table 2). These auxiliary functions encompassed specific features such as comparisons, analysis, and recommendations, along with capabilities for managing blood sugar, blood pressure, and sleep. Furthermore, it included features for nutrition assessment, questionnaire survey, dietitian consultation, access to a nutrition marketplace, generation of a nutrition report, monitoring of biochemical indicators, participation in nutrition-related social circles, and health assessment [19-21].

**Table 2.** WeChat miniprogram for nutrition management.

WeChat mini-program function		Miniprograms, n (%)
**Main function**		
	Food record/management/analysis/clock in	23 (85.2)
	Exercise recording/management/analysis/clocking	16 (59.3)
	Weight recording/management/analysis/clocking	12 (44.4)
	Nutrition class/video/popular science	19 (70.4)
**Auxiliary function**		
	Food list	4 (14.8)
	Food comparison	3 (11.1)
	Food inquiry/analysis	8 (29.7)
	Food/recipe recommendations	13 (48.1)
	Blood pressure/blood sugar/sleep management	7 (25.9)
	Nutrition assessment/questionnaire	8 (29.7)
	Nutrition expert consultation	13 (48.1)
	Nutrition mall	10 (37.0)
	Nutrition report	7 (25.9)
	Biochemical index	5 (18.5)
	Nutrition sharing/friend circle/community	10 (37.0)
	Health assessment	8 (29.6)

As depicted in Table 2, the average functional score across all miniprograms was 6 points, ranging from 2 points in diet assessment to 10 points in peppermint nutrition food. Among the analyzed miniprograms, 85.2% (23/27) of them offered diet management features, facilitating the recording of daily dietary intake to assess nutritional status; 70.4% (19/27) of them provided nutrition knowledge and classroom teaching functionalities; 59.3% (16/27) of them offered exercise management capabilities, enabling users to record their daily physical activities; and only 44.4% (12/27) of them incorporated weight management functionalities.

In the functional evaluation, we conducted a comprehensive evaluation of the 4 main functions of each miniprogram. Simultaneously, we scrutinized and evaluated the auxiliary features to ensure that users could access comprehensive nutrition management services. Through this comprehensive assessment, we aimed to furnish users with detailed feedback, assisting them in selecting a high-quality nutrition management miniprogram, which was tailored to their requirements. Furthermore, this endeavor aimed to enhance users’ health management practices and improve their overall and quality of life [22].

### uMARS Quality Rating

An overview of the engagement, functionality, aesthetics, and information scores for the top 5 and bottom 5 miniprograms was presented in Table 3. Among them, the highest-ranking miniprogram was the WeChat applet mint health, with a uMARS score of 3.88 (SD 0.73), followed by AI (artificial intelligence) dietary dietitian, chestnut food diary, peppermint nutritionist, and sannuo health in turn. Notably, the user score approached 4.6 and the functional scores were 7.0. In contrast, the lowest ranking was WeChat applet-nutritionist world, with a uMARS score of 2.85 (SD 1.09), followed by diet evaluation, peppermint nutrition pro, nutritional meal companion, and long light nutrition diet therapy. In addition, the participation, function, aesthetics, and information scores of miniprograms are summarized in Table 3.

**Figure 2.**
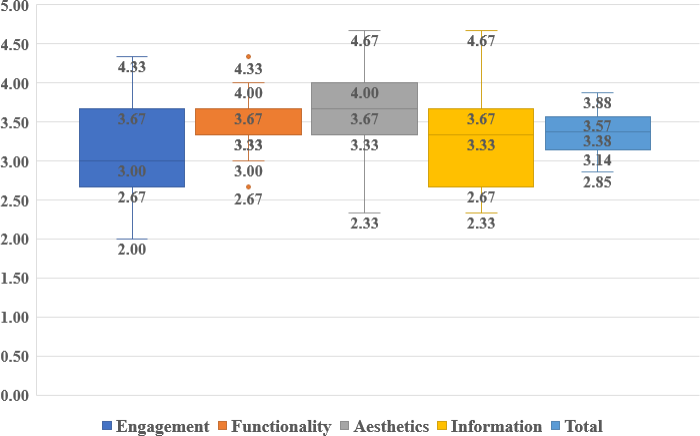
The User Version of the Mobile Application Rating Scale overall and section-specific scores of the nutrition management miniprograms (N=27).

The uMARS total quality median score, calculated on a scale of 5, was 3.38 (IQR 3.14-3.57), with an overall range from 2.85 to 3.88, indicating that most miniprograms achieved scores above 3 points. The uMARS score of engagement ranged from 2.00 to 4.33, with a median score of 3.00 (IQR 2.67-3.67). The functional dimension varied from 3.00 to 4.00, and the median score was 3.33 (IQR 3.33-3.67), with a lower score of 2.67 and a higher score of 4.33 outside the reference range. The aesthetic dimension spanned from 2.33 to 4.67, with a median score of 3.67 (IQR 3.33-4.00), and the informational dimension ranged from 2.33 to 4.67, with a median score of 3.33 (IQR 2.67-3.67; Figure 2). Detailed uMARS scores for all 27 miniprograms are provided in Multimedia Appendix 1.

**Table 3.** The 5 highest- and lowest-scoring miniprograms (N=27) based on the User Version of the Mobile Application Rating Scale (uMARS).

WeChat applet	Engagement score, mean (SD)	Functionality score, mean (SD)	Aesthetics score, mean (SD)	Information score, mean (SD)	uMARS score, mean (SD)
**Five highest-scoring miniprograms** ^a^					
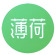	3.73 (0.96)	3.83 (0.72)	4.11 (0.33)	3.92 (0.67)	3.88 (0.73)
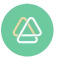	3.73 (0.88)	3.67 (0.49)	4.33 (0.50)	3.83 (0.58)	3.85 (0.68)
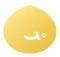	3.53 (0.92)	3.67 (0.49)	4.11 (0.60)	4.08 (0.79)	3.81 (0.76)
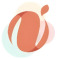	3.47 (1.13)	3.67 (0.49)	4.00 (0.50)	4.00 (0.60)	3.75 (0.79)
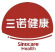	3.60 (1.12)	3.67 (0.65)	3.67 (0.50)	4.00 (0.74)	3.73 (0.82)
**Five lowest-scoring miniprograms** ^b^					
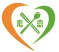	2.80 (0.86)	3.17 (0.58)	3.44 (1.01)	2.91 (0.79)	3.04 (0.82)
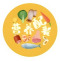	2.73 (1.03)	3.08 (0.79)	3.33 (0.50)	3.17 (0.94)	3.04 (0.87)
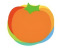	2.93 (1.22)	3.33 (0.49)	3.11 (0.93)	2.58 (1.24)	2.98 (1.04)
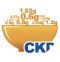	2.93 (1.16)	3.33 (0.49)	3.11 (1.05)	2.50 (1.17)	2.96 (1.03)
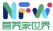	2.53 (1.89)	3.08 (0.90)	3.22 (1.09)	2.75 (1.14)	2.85 (1.09)

a Five highest-scoring miniprograms: (1) mint health, (2) AI (artificial intelligence) dietary dietitian, (3) chestnut food diary, (4) peppermint nutritionist, and (5) sannuo health.

b Five lowest-scoring miniprograms: (1) long light nutrition diet therapy, (2) nutritional meal companion, (3) peppermint nutrition pro, (4) diet evaluation, and (5) nutritionist world.

## Discussion

### Principal Findings

A single user rating may not accurately gauge the quality of the miniprograms, and some existing evaluation systems for nutrition management miniprograms lack a scientifically grounded approach to promoting human nutrition. At present, the field of nutrition management miniprograms in China is still in its nascent stage and requires continual refinement of technique [18]. Analysis of functional scores revealed that only 18.5% of the miniprograms achieved scores above 8 points, indicating a need for improvement in their nutrition-related functionalities and health care. Among the identified issues, concerns were raised regarding the accuracy and comprehensiveness of the food database, potentially resulting in access to inaccurate nutrition. Furthermore, most miniprograms lack personalized services, consequently failing to offer tailored nutrition advice based on users’ specific requirements and health conditions [23].

This study differs from prior research in several key aspects. First, many nutrition miniprograms now target specific disease types, tailoring diet management to the unique needs of patients. Second, some programs incorporate social circles to enhance user engagement, consequently fostering greater usage among patients [24]. Third, a subset of programs collaborates with the medical industry, engaging professional medical teams during development and establishing expert consultation platforms accessible via the WeChat miniprogram, tablet computer app, and computer software. Fourth, certain programs leverage data analysis and AI to deliver personalized nutrition advice and services, catering to individuals’ specific needs [25]. Fifth, the seamless integration of these programs within WeChat enables direct access without the need for installation or downloads, ensuring faster and more convenient processes. Finally, developers continuously refine and expand nutrition management programs in response to evolving technological advancements and user preferences, thus integrating new functionalities and enhancing user experience to elevate program quality and competitiveness [26]. Notably, our evaluation further revealed that the quality of nutrition management miniprograms varies significantly, with many exhibiting incomplete content, imperfect functionality, and limited individualization and intelligence [27].

### Limitations

Although this study reports some interesting and significant findings, there are several limitations associated with the use of WeChat miniprograms. First, nutrition miniprograms on WeChat are still undeveloped and nascent compared to nutrition apps with high usage rates. Second, due to the inability to download WeChat miniprograms, relevant data such as download counts and software size cannot be fully obtained, limiting our analysis of the data displayed on the platform [28]. Additionally, our research focuses solely on analyzing the functionality and quality of miniprograms within the WeChat platform, resulting in a relatively narrow scope. Accordingly, future studies could expand their scope by conducting questionnaire surveys among users of the existing miniprograms or by integrating user feedback more effectively [29]. Simultaneously, encouraging active involvement from health professionals in the development of mobile health apps is crucial for ensuring their effectiveness and relevance in promoting better health outcomes [30]. Moving forward, the trajectory of development should prioritize the enhancement of quality, introduction of innovative features, and fostering of active participation of professionals, consequently providing more scientifically grounded and practical personalized dietary guidance and enriching research on domestic nutrition management miniprograms [31-33].

### Conclusions

Our findings from the uMARS highlight a predominant emphasis on health aspects over nutritional specifications in the application of WeChat miniprograms related to nutrition management. The quality of these small programs is currently at an average level, with considerable room for functional improvements in the future.

## Supplementary material

10.2196/56486 Multimedia Appendix 1 uMARS (User Version of the Mobile Application Rating Scale) scale score.

## Abbreviations

AI: artificial intelligence
MARS: Mobile Application Rating Scale
uMARS: User Version of the Mobile Application Rating Scale
